# Supplementary material for: Health complexity assessment in primary care: A validity and feasibility study of the INTERMED tool
Source: PLoS One. 2022 Feb 18;17(2):e0263702. doi: 10.1371/journal.pone.0263702 (PMC8856552; doi:10.1371/journal.pone.0263702)
Supplement: S1 Table — (DOCX) [file pone.0263702.s001.docx]

| **S1 Table 1**. Profiles of the 230 PHC patients regarding INTERMED items and their clinical anchor points. | | | | | |
| --- | --- | --- | --- | --- | --- |
|  |  | **INTERMED clinical anchor point*** | | | |
|  | **Items** | **0** | **1** | **2** | **3** |
| **Biological** | Chronicity | 60 (26.1%) | 8 (3.5%) | 66 (28.7%) | 96 **(41.7%)** |
|  | Diagnosis dilemma | 67 (29.1%) | 116 **(50.4%)**** | 33 (14.3%) | 14 (6.1%) |
|  | Symptom severity | 74 (32.2%) | 62 (27.0%) | 88 **(38.3%)** | 6 (2.6%) |
|  | Diagnostic challenge | 79 (34.3%) | 124 **(53.9%)** | 24 (10.4%) | 3 (1.3%) |
|  | Complications and life threat | 149 **(64.8%)** | 61 (26.5%) | 20 (8.7%) | 0 (0.0%) |
| **Psychological** | Restriction in coping | 121 **(52.6%)** | 36 (15.7%) | 55 (23.9%) | 18 (7.8%) |
|  | Psychiatric dysfunction | 133 **(57.8%)** | 35 (15.2%) | 60 (26.1%) | 2 (0.9%) |
|  | Resistance to treatment | 165 **(71.7%)** | 57 (24.8%) | 6 (2.6%) | 2 (0.9%) |
|  | Psychiatric symptoms | 80 (34.8%) | 22 (9.6%) | 124 **(53.9%)** | 4 (1.7%) |
|  | Mental health threat | 102 (44.3%) | 105 **(45.7%)** | 23 (10.0%) | 0 (0.0%) |
| **Social** | Job and leisure problems | 138 **(60.0%)** | 45 (19.6%) | 36 (15.7%) | 11 (4.8%) |
|  | Social dysfunction | 171 **(74.3%)** | 22 (9.6%) | 22(9.6%) | 15 (6.5%) |
|  | Residential instability | 215 **(93.5%**) | 15 (6.5%) | 0 (0.0%) | 0 (0.0%) |
|  | Poor social support | 167 **(72.6%)** | 32 (13.9%) | 8 (3.5%) | 23 (10.0%) |
|  | Social vulnerability | 187 **(81.3%)** | 42 (18.3%) | 1 (0.4%) | 0 (0.0%) |
| **Health system** | Access to care | 111 **(48.3%)** | 50 (21.7%) | 69 (30.0%) | 0 (0.0%) |
|  | Treatment experience | 130 **(56.5%)** | 69 (30.0%) | 28 (12.2%) | 3 (1.3%) |
|  | Organization of care | 147 **(63.9%)** | 67 (29.1%) | 15 (6.5%) | 1 (0.4%) |
|  | Coordination of care | 159 **(69.1%)** | 28 (12.2%) | 13 (5.7%) | 30 (13.0%) |
|  | Health system impediments | 178 **(77.4%)** | 45 (19.6%) | 6 (2.6%) | 1 (0.4%) |

*Clinical anchor point: "0" – No vulnerability/only health education; "1" – Mild vulnerability/need for monitoring or prevention; "2" – Moderate vulnerability/need for treatment or inclusion in treatment plan; "3" -Severe vulnerability/need for immediate or intensive care

** Values in bold represent the highest percentage within the clinical anchor points
