# Supplementary material for: Health complexity assessment in primary care: A validity and feasibility study of the INTERMED tool
Source: PLoS One. 2022 Feb 18;17(2):e0263702. doi: 10.1371/journal.pone.0263702 (PMC8856552; doi:10.1371/journal.pone.0263702)
Supplement: S2 Table — (DOCX) [file pone.0263702.s002.docx]

| **S2 table 2.** Completeness of the INTERMED's domains in the health records. | | |
| --- | --- | --- |
| **Domain** | **INTERMED items** | **Availability in health records**  **(Frequency, %)** |
| **Biological** | Chronicity | 211 (88.7%) |
|  | Diagnosis dilemma | 202 (84.9%) |
|  | Diagnostic challenge | 194 (81.5%) |
|  | Complications and life threat | 136 (57.1%) |
|  | Symptom severity | 122 (51.3%) |
| **Psychological** | Resistance to treatment | 153 (64.3%) |
|  | Restriction in coping | 84 (35.3%) |
|  | Psychiatric symptoms | 81 (34.0%) |
|  | Mental health threat | 80 (33.6%) |
|  | Psychiatric dysfunction | 39 (16.4%) |
| **Social** | Job and leisure problems | 48 (20.2%) |
|  | Social dysfunction | 42 (17.6%) |
|  | Residential instability | 19 (8.0%) |
|  | Social vulnerability | 16 (6.7%) |
|  | Poor social support | 10 (4.2%) |
| **Health system** | Organization of care | 180 (75.6%) |
|  | Coordination of care | 78 (32.8%) |
|  | Health system impediments | 51 (21.4%) |
|  | Access to care | 25 (10.5%) |
|  | Treatment experience | 3 (1.3%) |
