# Supplementary material for: Health complexity assessment in primary care: A validity and feasibility study of the INTERMED tool
Source: PLoS One. 2022 Feb 18;17(2):e0263702. doi: 10.1371/journal.pone.0263702 (PMC8856552; doi:10.1371/journal.pone.0263702)
Supplement: Attachment — Submitted filename: Response to reviewers.docx [file pone.0263702.s003.docx]

## Dear Editor in Chief,

Dr. [Emily Chenette](https://everyone.plos.org/author/echenette/) .

Editor-in-Chief
PLOS One

Ref:

Title: Health Complexity Assessment in Primary Care: a validity and feasibility study of the INTERMED tool

Journal: PLOS One

Dear Dr. [Emily Chenette](https://everyone.plos.org/author/echenette/) ,

Thank you for the opportunity to resubmit our manuscript to the PLOS One. We would like to thank the reviewers’ comments and suggestions. They were very helpful and insightful, and we have incorporated them into the revised version of the manuscript.

The following are the changes we have made, and which have been highlighted in the current version of the manuscript.

**REF. EDITOR**

**Comment 1**: *“Please ensure that your manuscript meets PLOS ONE's style requirements, including those for file naming. The PLOS ONE style templates can be found at https://journals.plos.org/plosone/s/file?id=wjVg/PLOSOne_formatting_sample_main_body.pdf and* [*https://journals.plos.org/plosone/s/file?id=ba62/PLOSOne_formatting_sample_title_authors_affiliations.pdf*](https://journals.plos.org/plosone/s/file?id=ba62/PLOSOne_formatting_sample_title_authors_affiliations.pdf)*”*

Answer: The manuscript has been amended accordingly.

**Comment 2:** “In your Methods section, please provide a justification for the sample size used in your study, including any relevant power calculations (if applicable).”

Answer: We amended the text as requested by providing the following justification:

*“**Sample sizes varied greatly among previous psychometric evaluations of INTERMED, ranging from 30 [14] to 275 patients [21], with no guidance how to choose a value within that range. To determine that value, we followed the rule of thumb of at least five observations per each variable, aiming for a 10:1 ratio (10 patients per each of the 20 INTERMED items) [22,23], resulting in estimated 200 patients and their health care records. Assuming a dropout of 15% in a planned second wave of the collection (not reported here), we decided to recruit additional 30 people (depending on availability).* (p. 5, line 108 to 114).

**Comment 3:** *“We suggest you thoroughly copyedit your manuscript for language usage, spelling, and grammar. If you do not know anyone who can help you do this, you may wish to consider employing a professional scientific editing service*”.

Answer: Thank you for the suggestion. We have thoroughly reviewed the text.

**Comment 4:** *“We note that you have indicated that data from this study are available upon request. PLOS only allows data to be available upon request if there are legal or ethical restrictions on sharing data publicly. For information on unacceptable data access restrictions".*

Answer: The data set can be found here: <http://repositorio.uspdigital.usp.br/handle/item/263>

**Comment 5:** *“Please remove any funding-related text from the manuscript and let us know how you would like to update your Funding Statement. Currently, your Funding Statement reads as follows:”*

Answer: Thank you. We removed that sentence.

**REF. REVIEWER 1**

**Comment 1:** *“The abstract is hard to understand due to language problems”*

Answer: Thank you for the suggestion. We have thoroughly reviewed the text.

**Comment 2:** “The INTERMED has to be shortly explained – was it the interview or the questionnaire? What does it measure?”

Answer: Thank you for the helpful suggestion. We have included the following brief description:

*“Participants responded to INTERMED semi-structured interviews, scoring 20 variables in four domains (biological, psychological, social and health system).”* (p. 2, line 38 to 40)

**Comment 3:** *“The other assessment instruments have to be listed (methods).”*

Answer: Thank you for this helpful comment. We have listed and described all other instruments in the method section.

**Comment 4:** *“How is “health complexity” defined?”*

Answer: We have provided the following definition:

*“**Health complexity includes biological, psychological, social and health systems aspects. Having complex health needs is associated with worse outcomes and higher healthcare costs.”* (p. 2, line 30 to 32)

**Comment 5:** *“Please shorten the description of the use of specific correlation coefficients in the abstract”.*

Answer: Thanks for the suggestion. We have reviewed the description of the method and results in the abstract, as per suggestion.

**Comment 6:** “Spearman’s correlations located between 0.44 – 0.65”. Which correlations are reported? The INTERMED total score correlated with which variables to which amount? This must be described exactly and clearly. The same holds for the other correlations (or just name a few variables and their correlations – but use exact descriptions).”

Answer: Thanks for the suggestion. While reviewing this text, we have noticed some errors in reporting numerical data, which we have now addressed and amended the text as per suggestion:

*“Correlational analyses supported the validity of three INTERMED domains (social: Medical Outcomes Study Social Support Survey, rho= -0.44, psychological: Hospital Anxiety and Depression Scale, rho=0.58 and biological: WHO Quality of Life Brief - Biological, rho = -0.60), and the correlation between the total of INTERMED and the total of WHO Quality of Life Brief was rho= -0.59.”* (p.2, line 44 to 48)

**Comment 7:** *“7 minutes for an INTERMED interview is a very short time. How did this happen? (To be explained in the results section or discussion).”*

Answer: Thanks for this comment. We have provided an explanation in the discussion section, which read as follows:

*“The median of the length time (seven minutes) ranged between three and 32 minutes is compatible with the average PHC appointment duration in 39 countries. It is different from that found in specialized services, in which the INTERMED application time ranged from 20 to 40 minutes, with a smaller percentage of patients being considered non-complex [27].”* (p.19, line 353 to 357)

**Comment 8:** *“Please ask a native speaker to revise the manuscript”.*

Answer: Thank you for the suggestion. We have thoroughly reviewed the text.

**Comment 9:** *“Please insert line numbers in the revision”*

Answer: The line numbers have bene inserted.

**Comment 10:** *“identifying the epidemiology of patient complexity” – what is meant by this? The reference cited (9) does not refer to an epidemiological study.”*

Answer: Indeed, thank you for identifying this error. We have carefully reviewed all the references.

**Comment 11:** *“Please reduce the number of references considerably.”*

Answer: We have reduced the number of references.

**Comment 12:** ““how to effect the identification of complex patients” – I do not understand this sentence.”

Answer: The definition of complex patients is related to several factors, including the increase in the prevalence of chronic health conditions, both physical and psychiatric (comorbidities and multimorbidity), polypharmacy, excessive number of consultations and referrals to specialized follow-ups, social determinants of health (age, gender, lifestyle, social relationship) and identification of profiles and risk factors of the health conditions of the patient. We have defined the health complexity as follows:

‘*Health Complexity’ can be defined as “interference with the achievement of expected or desired health and cost outcomes due to the interaction of biological, psychological, social and health systems factors” [1]. That factor includes demographics, social capital, health and social experiences, and physical and mental health, which interact dynamically and non-linearly in an idiosyncratic manner for each individual [2–4].”* (p.4, line 77 to 79)

**Comment 13:** “The introduction must be re-written. It is not clear what the study wants to show. Perhaps the authors want to say that complexity is frequent in general practices but not so easy to detect? And that the INTERMED interview could be a useful assessment instrument for a GP. However, to date, only one study evaluated the usefulness and validity of the INTERMED interview in the frame of GPs. And, therefore, the present study aims to ….”

Answer: We rewrote the introduction to clarified what we want in ours study as per suggestion.

**Comment 14:** “Was the (already recruited) sample divided into groups or the sampling based on various age groups?”

Answer: We created a table for each health unit, in which the sample was divided into gender and subdivided into age groups. We have provided a clear description of our approach, which reads as follows:

 “*To maximise the chance of gathering representative data, we deployed a quota sampling method, by dividing the population into gender and age groups (18-30, 31-40, 41-50, 51-60 and ≥60 years) [24]. Within each group, a convenience sample was selected.*”. (p. 5, line 113 to 116)

**Comment 15:** *“Instruments: The description of the INTERMED is hard to understand. Perhaps the authors could insert a Figure showing the INTERMED grid. The INTERMED interview assesses four domains: biological, psychological, social, and health care use. In each domain, five variables are rated on a scale ranging from 0-3, resulting in 20 scores. Each score ranges from zero evidence for or health service need (0), to a clear and serious disturbance or health service needs (3).”*

Answer: We have added the INTERMED GRID (Figure 1) and added the following description:

“*The INTERMED is a semi-structured interview that synthesizes data from four health-related aspects (domains): 1) biological, 2) psychological, 3) social, and 4) health system, assessed in the context of time (history, current state, and vulnerability/prognosis) [11,14,25]. Within each of the four domains, there are five specific variables (items), totaling 20. Each item has specific clinical anchor points described, ranging between 0 (no vulnerability / only health education) to 3 (severe vulnerability /need for immediate care or intensive) [25] (Fig 1).”* (p.6, line 133 to 138)

**Comment 16*:*** *“There are 16 lead questions for the interview. The remaining 4 items are prognostic items – that is, the interviewer gives a prognostic score (without a lead question).”*

Answer: The prognostic item of each domain does not involve a direct question for a patient. Instead, an interviewer would score that item using their clinical knowledge. The interviewer would then analyse the responses for each domain and score the prognosis according to his/her best judgment. We amended the text to clarify this:

*“Based on the information obtained from the answers to the 16 questions mentioned, the health professional assesses the four items of prognosis/vulnerability (one for each domain). A health professional is free to follow the topic guide, or skip or modify specific questions according to what a patient said spontaneously.”* (p.7, line 145 to 149)

**Comment 17:** *“Why was Spearman’s correlation coefficient used? Why not Pearson for the correlation between INTERMED total score and HADS score, for instance?”*

Answer: We used Spearman’s correlation because in the study population, INTERMED correlations with HADS, MOSS WHOQOL and CCI did not meet the assumption of normality, required for Pearson's correlation. We changed the sentence to.

"We performed the Shapiro-Wilk test to examine the distribution of INTERMED data associated with the other four instruments (i.e. HADS, MOSS-SSS, WHOQOL, and CCI). The results did not meet the prerequisites for normality and homogeneity, so it was decided to perform Spearman's correlation analysis. Spearman coefficients (rho) ranging from 0.10 to < 0.40, from 0.40 to < 0.70, from 0.70 to < 1.00, were interpreted as weak, moderate and strong respectively (39).” (p. 10, line 222 to 227)

**Comment 18:** *“What is meant by Pearson X2? Is it a chi-squared test? What is compared here? A chi-squared test compares groups with dichotomous outcomes. Were Pearson correlation coefficients calculated? Please clarify. The distribution of health care use is probably skewed. Here, a Pearson correlation is probably not the best choice.”*

Answer: We agree with the comment. As per suggestion, we redid the calculations and modified the text accordingly:

" Hierarchical multiple linear regression analysis was used to test if ‘health system’ use could predict INTERMED-based biopsychosocial complexity. *One dependent variable was entered into the model (i.e., continuous INTERMED scores) and the three independent dichotomous variables (i.e., PHC, non-PHC, and medication use). To determine the best predictive model, we performed an analysis in three stages: first, we decided to build the model through the forward selection using the AIC as a criterion, and adjusting two categorical variables - age and sex and then; second, graphical analysis of the residual outliers influents to remove discrepant effects on the predictive results of the regression model, using the same AIC criterion [37,38];* *third, we analysed the Shapiro-Wilk test to performed a diagnosis of the model to confirm that the withdrawal of the outliers influents was consistent with the normality requirement of the regression model*.” *(*p.11, line 229 to 239)

**Comment 19:** *“How many patients refused to participate? What are the differences between non-responders and responders? What were the reasons to refuse?”*

Answer: “*We invited two hundred and forty-three (243) patients, of whom five didn't agree to participate, and two hundred and thirty-eight (238) agreed. Eight people were excluded though as they chose not to complete all questionnaires. Table 1 shows socio-demographic characteristics of the 230 participants [mean age = 45.92 (±15.43) years, 56.1% female, 53.5% reported being white, 43.5% reported incompleted or completed high education and 40.4% were employed].”* (p.12, line 258 to 263)

**Comment 20:** *“Validity: The correlations ranged from 0.44 to 0.65? In Table 3, there are smaller correlations presented*”

Answer: We thank you for this helpful comment. We spotted some errors in reporting numerical data, which we addressed and we amended the text as follows:

"*With regards to concurrent validity, using Spearman's correlation coefficients, between INTERMED and HADS, MOS-SSS, CCI, and WHOQOL-BREF scores, we can determined that there was moderate correlations between the total INTERMED score and its psychological domain with HADS (ranging from 0.501 to 0.587, p <0.001);  the INTERMED social domain and MOS-SSS (-0.443, p <0.001); the INTERMED psychological domain with all WHOQOL domains and their total score (ranging between 0.439 and 0.586, p< 0.000); and the INTERMED biological domain with the physical domain of WHOQOL (-0.585, p<0.000) (Table 3). All results remain significant after controlling for the type 1 error in the set of comparisons, except for MOS - SSS for health system.”* (p.14, line 278 to 286)

**Comment 21:** *“I do not understand Table 4. In Table 4, chi-squared statistics and p-values are presented. What was calculated here? Which groups were compared? Were Pearson correlations calculated? Then the correlation coefficients should be shown, together with a p-value.”*

*Answer:* We agree, it was unclear. We came to a conclusion that to address it, we would have to modify our approach to calculating the association between INTERMED, and the use of the health service. For that we used hierarchical multiple linear regression. Table 4 and the text were amended, the latter now reads as follows:

*“**To verify whether the ‘health system’ use (PHC, non-PHC, and medication) can predict patients' levels of complexity based on the INTERMED criterion, we used hierarchical multiple linear regression. The analysis resulted in a model 1 [F(3,226) = 14.1 p < 0.001, R2 = 0.158, AIC = 1552] and after adjustment of confounders, in a model 2 [F(8,221) = 10.2, p < 0.001, R2 = 0.243, AIC = 1529]. The results of the Shapiro-Wilk test were 0.981 (p = 0.003), and 0.982 (p = 0.005) respectively, meaning that the assumptions of normality were not met and, therefore, models 1 and 2 were inadequate. Next, through graphical analysis, we identified outliers. These were patients who regardless of the complexity level, either used the health system sporadically (only in A&E) or used the system in an exaggerated way (with excessive consultations in A&E, plus 10 consultations in PHC, and more than 10 medications). After analyzing and excluding these residual outliers influents, a model 3 was run [F(8,209) = 16.8, p < 0.001, R2 = 0.391, AIC = 1380], for which the result of Shapiro-Wilk was 0.996, p = 0.776, meaning the normality assumption was met and the model 3 was adequate. Overall, after adjusting for confounders and excluding the 12 residual outliers influents (model 3), the use of PHC, non-PHC, and medication were predictors of complexity according to the INTERMED criteria. Table 4 shows the development of the model.” (*p.15, line 300 to 315)

**Comment 22:** *“Please shorten the paragraph regarding “feasibility”*

Answer: Thank you for the suggestion. We shorten the said paragraph.

**Comment 23:** *“The discussion must be shortened. Some parts are hard to understand due to language”*

Answer: Thank you. We shorten the discussion section and reviewed the text.

**REF. REVIEWER 2**

**Comment 1:** *“Is the Pearson chi square test appropriate when one variable is a parametric test score (i.e., scores on the INTERMED current status)?”*

Answer: Thank you. Please see the response to Comment 18 from Reviewer 1.

**Comment 2:** *“Why was Spearman rho used as the correlation coefficient? Were the data in question rank ordered?”*

*Answer: Thank you.* Please see the response to Comment 17 from Reviewer 1

**Comment 3:** *“Is coefficient alpha appropriate for an instrument that is not tau equivalent? That is, can it be demonstrated that the INTERMED is an unidimensional instrument appropriate for the alpha coefficient?”*

*Answer:* Thank you for this comment. We agree. INTERMED isn’t unidimensional neither a tau equivalent, thus we recalculated using the omega coefficient. We rewrote the method section accordingly:

*“We performed internal consistency analysis to assess reliability of the INTERMED. Factor analysis was conducted to determine the unidimensionality and tau-equivalence of INTERMED. Next, we determined the best calculation for the internal consistency would be Omega Coefficient and values ranging from <0.5, from 0.5 to 0.6, from 0.6 to 0.7, from 0.7 to 0.8, from 0.8 to 0.9 and from ≥ 0.9, were interpreted as unacceptable, poor, questionable, acceptable, good and excellent the same used in Alpha Coefficient [39].*” (p.11, line 241 to 246)

As well as the results section: *“The data proved to be adequate to perform the factor analysis (KMO = 0.78, Bartlett sphericity test, x (190, n = 230) = 1512, p = <0.001). The factor analysis suggested the extraction of four factors. Thus, INTERMED does not meet the one-dimensionality and tau-equivalence prerequisites (ranking of the factorial load = 0.11 - 0.8). Omega coefficient was 0.834, suggesting a good internal consistency [39,40]. After deleting each of the 20 items from INTERMED, omega coefficient values' ranking ranged from 0.817 to 0.836. Four items showed no decrease in the original omega coefficient value when deleted: ‘treatment experience’ (0.835); ‘resistance to treatment’ and ‘access to care’ (both measure 0.836); and ‘job and leisure problems’ (0.837).”* (p. 15, line 291 to 299)

**Comment 4:** *“Why was the term "construct validity" used on page 9?”*

*Answer:* We agree we meant ‘convergent validity’.

**Comment 5:** *“What is an "academic clinician" (page 9)?”*

Answer: Academic clinician are clinically active health care professional who also work as researchers and lecturers. We removed this expression, to avoid any misunderstandings.

**Comment 6:** *“On page 7 is the following statement: "we evaluated the concurrent validity of each of its four domains with other well-validated specific instruments for these domains." The next sentence starts listing the other instruments, the first of which is the Socio-demographic Questionnaire that was "developed for the study." Is the assertion that this instrument was "well-validated?" Or, the adaptation resulting in the Questionnaire for Evaluation of Health Services Use? These scales were not specifically used in the computation of the validity coefficients, but nevertheless there does seem to be a gap between the Term "well-validated" and documentation thereof, including documentation of the validity for criterion variables HADS, MOS-SSS, CCI, and WHOQOL-BREF.”*

*Answer:* We agree with the comment. For the validation of INTERMED we used well-validated instruments such as HADS, MOS-SS, CCI and WHOQOL. The instruments we developed for the study were sociodemographic, applicability and use of the health service, and were used in another stage of the research, such a description of the population characteristic, understanding of the satisfaction about the applicability of INTERMED by patients and analysis of the use of the service associated with INTERMED.

Please see our response to comment 3 of the reviewer 1.

**Comment 7:** *“Was consideration given to the control of type 1 error in the series chi square tests, and Spearman correlations? If not, why not?”*

*Answer:* Thank you for the question. We are aware that classicists argue that correction for multiple testing is mandatory. However, the p values presented in table 3 for each domain are almost all less than 0.000, that is, at most 0.0005. For the 9 comparisons together, the Bonferroni technique would give the final value of p in the adjusted tests, p = 0.005. All the results would remain the same, except for the correlations between MOS - SSS and the health system domain with p = 0.013. Therefore, we took an epidemiologist and rationalist approach, and argued that in this case the Bonferroni adjustment defied common sense and increased type II errors.

Rothman, K. J. (1990). "No Adjustments Are Needed for Multiple Comparisons." Epidemiology 1(1): 43-46.

Once again, thank you for their time and comments on this manuscript.

Sincerely,

Camila Almeida de Oliveira
