# Supplementary material for: Health complexity assessment in primary care: A validity and feasibility study of the INTERMED tool
Source: PLoS One. 2022 Feb 18;17(2):e0263702. doi: 10.1371/journal.pone.0263702 (PMC8856552; doi:10.1371/journal.pone.0263702)
Supplement: Attachment — Submitted filename: renamed_2f54d.docx [file pone.0263702.s004.docx]

## Dear Editor in Chief,

Dr. [Emily Chenette](https://everyone.plos.org/author/echenette/) .

Editor-in-Chief
PLOS One

Ref:

Title: Health Complexity Assessment in Primary Care: a validity and feasibility study of the INTERMED tool

Journal: PLOS One

Dear Dr. [Emily Chenette](https://everyone.plos.org/author/echenette/),

Thank you for the opportunity to resubmit our manuscript to the PLOS One. We would like to thank the reviewer’ comments and suggestions. They were very helpful and insightful, and we have incorporated them into the revised version of the manuscript.

The following are the changes we have made.

The changes in the current version of the manuscript have been highlighted.

**REF. REVIEWER 1**

**Comment 1:** The manuscript has been revised and the language has improved. However, I still have the impression that the manuscript was not edited by a native speaker (e.g. expressions such as “18.3 % of the patients were classed as complex” in the abstract or “This study aimed to assess… to assess…). Also, for instance, line 232: “Patients were excluded if they were determined to be unable to consent. In my opinion, the language of the manuscript must be improved.

Answer: Thank you for the suggestion. We used the Editage website (recommended by the journal) to reviewed the manuscript

**Comment 2:** *“In the Methods Section of the Abstract the cut-off for complexity must be mentioned”.*

Answer: Thank you for the suggestion. We have included the cutoff in the abstract.

**Comment 3:** “*The Methods Section of the Abstract must be re-written. Example for a better formulation: Biopsychosocial health care needs were assessed by using the INTERMED interview… Participants with a total INTERMED score >20 were classified as complex / having complex health care needs. Quality of life was measured by …”*

Answer: Thank you for the suggestion. We have thoroughly reviewed the text.

**Comment 4:** *“The Results Section of the Abstract should be re-phrased. Example: Results showed a high correlation between INTERMED total score (not “whole” score) and quality of life (r=0.59).
• The above formulations are just examples. The next paragraphs also lack scientific precision. I would really ask the authors to scan other scientific papers and use appropriate formulations. The whole study decreases in value if the expressions are not precise and adequate.
• T- and beta-values should be rounded to two decimal points (instead of three).
• the sentence “In a multiple linear regression analysis, the use of PHC (β = 2.121, t = 2.100, p=0.037), services other than PHC (β = 3.052, t = 3.970, p<0.001) and medication (β = 3.654, t = 4.164, p<0.001) predicted the INTERMED score.” is hard to understand. Perhaps a change to “ the use of PHC (), any other health care service (than PHC) () and the use of any medication () were associated with a higher INTERMED score.”*

Answer: Thank you for the suggestion, we agree with you and we have thoroughly reviewed the text.

**Comment 5:** *“I do not understand the variable “PHC use”. All the patients were recruited in a primary health care setting. So, everybody score a 1 on this variable?”*

Answer: The PHC variable is related to appointments scheduled in the last six months before the survey, excluding the interview day. The interviewed patients were not necessarily at the health unit for consultations: they were, in some cases, for dispensing medication or making an appointment, or even responsible for another patient. We rewrite lines 139 to 141 and lines 206 and 207 for better understanding.

**Comment 6: “**The introduction must be revised by a good native speaker”

Answer:

**Comment 7:** *“Sampling technique: I do not understand the “rule of thumb” – five observations per variable. The validity of the INTERMED was measured by using the total score (and not by calculating correlations with each item).”*

Answer: The” rule of thumb” of five observations per variable was not used. It was only a way do initially discuss and reach a final goal for sample size. That becomes very clear when we say that ***a final sample size of 230 was used.*** Literature points to the fact that our sample size is comfortable for reaching our goals:

See for instance: (Hand, Chris. (2017). Re: Justifying sample size in EFA?. Retrieved from: <https://www.researchgate.net/post/Justifying-sample-size-in-> EFA/58e7afb65b4952320415e46d/citation/download.)

“An alternative rule of thumb for EFA is based on the sample size and the number of variables to be included.  A minimum would be five times as many observations as you have variables, but a more acceptable ratio is 10:1.  You might find Hair, Black, Babin and Anderson's book Multivariate Data Analysis helpful”

Hair, Black, Babin & Anderson. Multivariate Data Analysis: Global Edition. 2010. Pearson Education. ISBN-13: 9780135153093

“Although determination of appropriate sample size is a critical issue..., there is no consensus in the literature regarding what would be the appropriate sample size for SEM. Some evidence exists that simple SEM models could be meaningfully tested even if sample size is quite small (Hoyle, 1999; Hoyle and Kenny, 1999; Marsh and Hau, 1999), but usually, *N* = 100–150 is considered the minimum sample size for conducting SEM (Tinsley and Tinsley, 1987; Anderson and Gerbing, 1988; Ding, Velicer, and Harlow, 1995; Tabachnick and Fidell, 2001). Some researchers consider an even larger sample size for SEM, for example, *N* = 200 (Hoogland and Boomsma 1998; Boomsma and Hoogland, 2001; Kline, 2005). Simulation studies show that with normally distributed indicator variables and no missing data, a reasonable sample size for a simple CFA model is about *N* = 150 (Muthén and Muthén, 2002). “

Source: Kamel, Mahmoud. (2017). Re: What is the minimum sample size for structural equation modelling?. Retrieved from: [*https://www.researchgate.net/post/What-is-the-minimum-sample-size-for-structural-equation-modelling/598b750feeae3900a72d9a5d/citation/download*](https://www.researchgate.net/post/What-is-the-minimum-sample-size-for-structural-equation-modelling/598b750feeae3900a72d9a5d/citation/download)*.*

***Our Note: SEM = Semi Estructal Equations, a model even more restrictive than ours for the matters of validation.***

**Comment 8:** *“Sampling technique: The authors state that previous INTERMED studies used different sample sizes. These studies varied widely in object and settings. So, why should there be a common sample size calculation for all these studies? An INTERMED study does not need specific assumptions for a sample size calculation (compared to other validation studies). For a validation study one can, for instance, calculate the appropriate sample size based on the assumed correlation between the main instrument (INTERMED) with the gold standard with which I compare the instrument. However, very few validation studies do a power calculation prior to the beginning (see for instance* [*https://hqlo.biomedcentral.com/articles/10.1186/s12955-014-0176-2*](https://hqlo.biomedcentral.com/articles/10.1186/s12955-014-0176-2)*. One could also just describe that the sample was collected consecutively over a specific time period – if this was the case – resulting in n=230 patients.”*

Answer: Thank you for the suggestion. We have thoroughly reviewed the text. See the answer of the comment 7

**Comment 9:** *“Line 310: Please change “The instruments were administered between November 2018 to June 2019.” Into “**The study was conducted between November 2018 to June 2019” or “Patients were included between November 2018 to June 2019” – or something similar. Please adjust other formulations to a more scientific language.”*

Answer: Thank you for the suggestion. We have thoroughly reviewed the text.

**Comment 10:** *“Line 293: The patients were recruited in a PHC setting. So, each patient scored a 1 on the variable “PHC use”?”*

Answer: Patients scored with "1" if they had been in an appointment in the last six months before the survey, excluding the day of the interview.

**Comment 11:** *“Line 343: adjusting for sex and gender – or controlling for…”*

Answer: Thank you for the suggestion. We have thoroughly reviewed the text.

**Comment 12:** *“Lines 344-346: This is not to understand. Outliers are removed beforehand (when data appears to be absolutely implausible). Also, the language is difficult to understand. What is meant by “influent”? How many data was withdrawn from the sample? I would recommend to skip step 2 and 3.”*

Answer: In Linear Regression, there are leverage outliers, which move away from the central points. When we have a leverage point that influences the slope of the regression line, it is called influential points. To detect these points, we must perform an analysis of outliers to reduce the influence on the model.

"It is well known that inferences based on least squares regression can be strongly influenced by only a few observations in the data, and the model may reflect unusual features of those observations rather than the overall ones between the variables" (Zakaria, 2014).

The article can be helpful: Zakaria, Arimiyaw. "On the Detection of Influential Outliers in Linear Regression Analysis." American Journal of Theoretical and Applied Statistics 3, No. 4 (2014): 100. https://doi.org/10.11648/j.ajtas.20140304.14.

To reach an adequate model is necessary to analyze and remove influential outliers. Firstly, performing a model with the outliers to obtain the R2; second, followed the graphic analysis of the outliers to determine their influence (through the Coefficient of Determination Ratio); third, perform the model again without the influential points and compare the R2 values. In case of modification of the R2, the new model is adequate.

See, for instance: Sall, John. "Multiple Regression Residual Analysis and Outliers." Accessed August 18, 2021. https://www.jmp.com/en_us/statistics-knowledge-portal/what-is-multiple-regression/mlr-residual-analysis-and-outliers.html.

**Comment 13:** *“Line 351: Why was a factor analysis conducted? I would recommend to omit this procedure. It is not part of the study aim. Also, in my opinion, the INTERMED is not uni-dimensional. It consists of four different domains.”*

Answer: Thank you for the suggestion. We have thoroughly reviewed the text.

**Comment 14:** *“Descriptive statistics should be described first. Followed by the more complex statistics.”*

Answer: Thank you for the suggestion. We have thoroughly reviewed the text.

**Comment 15:** *“Table 2 should be omitted or changed in a* *Supplemental Table”*

Answer: Thank you for the suggestion. We have thoroughly reviewed the text.

**Comment 16*:*** *“Please round correlation and p-values to two decimal points.”*

Answer: Thank you for the suggestion. We have thoroughly reviewed the text.

**Comment 17:** *“Line 497: What means “after controlling for type 1 error?””*

Answer: Thank you for the suggestion. We have thoroughly reviewed the text.

**Comment 18:** *“Lines 503-505: The INTERMED is not assumed to be uni-dimensional. Somatic issues (for instance) are not on the same dimension as psychological issues. Perhaps the factor analysis should be omitted. It is also not clear, which factor analysis has been conducted.”*

Answer: Thank you for the suggestion. We have thoroughly reviewed the text.

**Comment 19: *“****The outliers should not be excluded. These are the patients who use the system excessively (or not at all). There is no reason to exclude them. In contrast, the patients with the highest health care use are probably those with the highest INTERMED scores.”*

Answer: Thank you for the suggestion. See the answer of comment 12. After analyzing the influential outliers, we observed that these patients do not present a pattern of the level of complexity. Both patients who do not use the service and patients who use it exaggeratedly have a considerable variation between levels of complexity.

Some examples:

1) patient who went to the U&E due to asthma, took 13 medications, and was referred to the Psychosocial Support Center - INTERMED scores 11 points (no complexity).

2) patient used urgent and emergency services because of low back pain and an appointment at the PHC. – INTERMED score 28 points (complex).

These patients do not follow the pattern of use of the service concerning the complexity level, influencing the prediction of the model. In this way, we chose to exclude the influential outliers to have a suitable model for our reality.

**Comment 20:** “Table 5 must be omitted.”

Answer: Thank you for the suggestion. We have thoroughly reviewed the text.

**Comment 21: *“****line 627 ff: a median does not range.* *The range of the time was 3-32, the median was 7.”*

*Answer:* Thank you for the suggestion. We have thoroughly reviewed the text.

**Comment 22: “**Lines 637 ff: The cut-off could be investigated with the present study data. One could run an ROC analysis with different cut-offs for complexity and look at the sensitivity and specificity of the various cut-offs regarding a specific outcome (this would need a categorization of the patients according to an external criterion). So, the authors do not have to do this. But a clustering method is not the method to investigate the appropriateness of a cut-off.”

Answer: Thank you for the suggestion. We have thoroughly reviewed the text.

Once again, thank you for their time and comments on this manuscript.

Sincerely,

Camila Almeida de Oliveira
