# Supplementary material for: Health complexity assessment in primary care: A validity and feasibility study of the INTERMED tool
Source: PLoS One. 2022 Feb 18;17(2):e0263702. doi: 10.1371/journal.pone.0263702 (PMC8856552; doi:10.1371/journal.pone.0263702)
Supplement: Attachment — Submitted filename: renamed_26913.docx [file pone.0263702.s005.docx]

## Dear Editor in Chief,

Dr. [Emily Chenette](https://everyone.plos.org/author/echenette/) .

Editor-in-Chief
PLOS One

Ref:

Title: Health Complexity Assessment in Primary Care: a validity and feasibility study of the INTERMED tool

Journal: PLOS One

Dear Dr. [Emily Chenette](https://everyone.plos.org/author/echenette/),

Thank you for the opportunity to revise our manuscript. We would also like to thank the reviewer 1 for their comment. It was very helpful and insightful. Below we describe how it has been addressed in the revised version of the manuscript.

**REF. REVIEWER 1**

**Comment 1:** It is stated that the health records of the patients were reviewed. However, it is not clear if the INTERMED interview scores were changed based on this reviews? Or how the information gained in these reviews was transferred to the data used in the analysis. Or what exactly was done with the information gained in the health records reviews (in terms of changing interview scores).

Could you please explain this - and if the INTERMED interview scores were changed- provide information about these changes (did the total scores increase after the review, the amount of change (mean values before the reviews, mean values after the reviews etc.)?
This would provide an insight about the information that is missing when applying the INTERMED interview.

*Answer:* Thank you for this thoughtful comment.

The purpose of analyzing the health records was to understand whether health care professionals might be able to find, in existing patient health records, information required to assess biopsychosocial complexity of needs. If that was the case, the INTERMED interview may be redundant. As such, this step served the purpose of evaluating an aspect of feasibility, that is the tool’s practical applicability to the real world. We have clarified this in the text *(please see – in ‘all markup view’- lines 212 to 216* and *222*).

We confirm that health records were reviewed after the instruments were applied (*please see line 210*). We have now also explicitly stated in the text that the interview scores were not influenced by the data obtained from the health records review *(please see lines 217 -218).*

We have found that over 50% of ‘biological’ domain data could be found in the existing health records, but data related to ‘psychological’, ‘social’, and ‘health system’ was scarce *(please see lines 320 to 321).* This way we have demonstrated that the INTERMED interview may be worthwhile clinician’s time as it captures new and needed information (*i.e.,* it is practically applicable).

We have concluded that the INTERMED interview can assist health professionals in obtaining new useful information for biopsychosocial assessment and care planning, in the primary health care context, as such it may be worthwhile clinician’s time *(see lines 345 to 351).*

Once again, thank you for the comments on this manuscript.

Sincerely,

Camila Almeida de Oliveira
